# Edge/Fog Computing Technologies for IoT Infrastructure II

**DOI:** 10.3390/s23083953

**Published:** 2023-04-13

**Authors:** Taehong Kim, Seong-eun Yoo, Youngsoo Kim

**Affiliations:** 1 School of Information and Communication Engineering, Chungbuk National University, Cheongju 28644, Republic of Korea; taehongkim@cbnu.ac.kr; 2 School of Artificial Intelligence, Daegu University, Gyeongsan 38453, Republic of Korea; seyoo@daegu.ac.kr; 3 Department of Artificial Intelligence, Jeonju University, Jeonju 55069, Republic of Korea

The prevalence of smart devices and cloud computing has led to an explosion in the amount of data generated by IoT devices. Moreover, emerging IoT applications, such as augmented and virtual reality (AR/VR), intelligent transportation systems, and smart factories, require ultra-low latency for data communication and processing. Edge/fog computing is a new computing paradigm where fully distributed fog/edge nodes located near end devices provide computing resources. By analyzing, filtering, and processing at local fog/edge nodes instead of transferring tremendous amounts of data to centralized cloud servers, fog/edge computing can significantly reduce processing delay and network traffic. With these advantages, fog/edge computing is expected to be one of the key enabling technologies for building IoT infrastructure.

In order to examine the recent research on edge/fog computing technology for building IoT infrastructure, this Special Issue published seven articles after a thorough review process. The selected articles cover diverse topics such as the distributed orchestrator model, task offloading, task scheduling, and mutual authentication protocol on edge/fog computing infrastructure, which represent the recent trends and the state-of-the-art algorithms of edge/fog computing technologies.

The article “Distributed Agent-Based Orchestrator Model for Fog Computing” by Agnius Liutkevičius et al. [1] presents a distributed agent-based orchestrator model that enables flexible service provisioning in a dynamic fog computing environment, accounting for the resource constraints and security levels of fog nodes. Unlike previous works that rely on a central control entity, the proposed model distributes decision-making agents across every fog node, promoting efficient decision-making, energy conservation, reduced delay, and improved system performance by placing decision-making at the closest fog node. The proposed model is implemented and tested on real hardware as a prototype, demonstrating its superior performance in terms of response latency and computational overhead when compared to the batch algorithm.

The article titled “Dynamic Task Offloading for Cloud-Assisted Vehicular Edge Computing Networks: A Non-Cooperative Game Theoretic Approach” [2] presents an efficient dynamic task offloading scheme based on a non-cooperative game (NGTO) to overcome the challenges of determining a real-time processing location for offloaded tasks in the vehicular edge computing (VEC) environment, where high vehicle mobility and overload problems are prevalent. The authors propose the best response offloading strategy that dynamically adjusts the task-offloading probability between the multi-access edge computing (MEC) server and the cloud to maximize utility for each vehicle. To design the VN (vehicular network) model, they considered the movement of vehicles at different speeds and utilized the vehicle-to-RSU (V2R) communication mode for local road-side units (RSU) computing. Vehicles can offload their computing tasks via a base station (BS) or RSUs. Through EdgeCloudSim simulations, the proposed scheme achieved performance guarantees, reduced response time, and task failure rates.

The next article, “Collaborative Task Offloading and Service Caching Strategy for Mobile Edge Computing” by Xiang Liu et al. [3], focuses on the collaborative task offloading problem assisted by a dynamical caching strategy in mobile edge computing (MEC) and proposes a two-layer computing strategy called joint task offloading and service caching (JTOSC) to effectively reduce the maximum delay of all users. The outer layer in JTOSC iteratively updates the service caching decisions using Gibbs sampling, while the inner layer adopts a fairness-aware allocation algorithm and an offloading revenue preference-based bilateral matching algorithm to obtain a better computing resource allocation and task offloading scheme. The simulation results indicate that the proposed strategy outperforms existing methods in terms of maximum offloading delay, service cache hit rate, and edge load balance.

The article “Local Scheduling in KubeEdge-Based Edge Computing Environment” by S. Kim and T. Kim [4] investigates the performance of KubeEdge in terms of computational resource distribution and latency between edge nodes. The study reveals that forwarding traffic between edge nodes leads to degraded throughput and increased service delay in an edge computing environment. To mitigate this problem, the authors propose a local scheduling scheme that processes user traffic locally at each edge node. The performance evaluation demonstrates that the local scheduling scheme outperforms the existing load-balancing algorithm in KubeEdge in an edge computing environment. 

The following article, “Latency-Aware Task Scheduling for IoT Applications Based on Artificial Intelligence with Partitioning in Small-Scale Fog Computing Environment” [5], proposes a task scheduling algorithm that uses artificial neural networks with partitioning capabilities to enable multiple edge servers to learn and calculate hyperparameters in parallel. This approach is designed to reduce scheduling time and improve service level objectives while minimizing energy consumption in small-scale fog computing environments. The performance evaluations demonstrate that the proposed algorithm outperforms existing scheduling algorithms in terms of latency reduction and energy consumption.

The article “Autonomous Mutual Authentication Protocol in Edge Networks” [6] addresses the challenge of designing a mutual authentication protocol for the large number of autonomous devices in edge networks. To overcome this, the authors propose a novel decentralized protocol based on a public key system, octet-based balanced-tree transitions, challenge–response mechanism, device unique ID, pseudo-random number generator, time stamps, and event-specific session keys. The protocol is analyzed for its effectiveness against typical attacks, and its feasibility and effectiveness are demonstrated through experimental analysis in a real-world scenario with various edge devices.

The next article, “Cooperative Downloading for LEO Satellite Networks: A DRL-Based Approach” [7], addresses the challenge of transmitting collected data to ground stations in low Earth orbit (LEO) satellite-based applications, where high satellite mobility results in insufficient download time. To address this issue, the authors propose a deep reinforcement learning (DRL)-based cooperative downloading scheme that formulates a Markov decision problem (MDP) with the objective of maximizing the downloaded data. They adopt a soft actor critic (SAC)-based DRL algorithm to learn the optimal approach. The evaluation results demonstrate that the proposed scheme achieves a higher utilization of the satellite’s contact time compared with other schemes, making it an effective approach for LEO satellite-based applications.
